# An Unexpected Pacemaker Response to Catheter Ablation: Failure of Pacing Pulse Delivery During Asynchronous Pacing Mode

**DOI:** 10.19102/icrm.2023.14073

**Published:** 2023-07-15

**Authors:** Laith Alkukhun, Peter M. Jessel, Khidir Dalouk, David J. Shim, Merritt H. Raitt, Ignatius Gerardo E. Zarraga

**Affiliations:** ^1^VA Portland Health Care System, Portland, OR, USA; ^2^Knight Cardiovascular Institute, Oregon Health & Science University, Portland, OR, USA

**Keywords:** Ablation, asynchronous pacing, electromagnetic interference, failure to capture, lead–tissue interface, pacemaker

## Abstract

Radiofrequency (RF) ablation can be a source of electromagnetic interference (EMI) for cardiovascular implantable electronic devices (CIEDs). The response of CIEDs to this type of EMI can be variable and unpredictable. We report a case with an uncommon response where there was a failure to deliver pacing pulses to both atrial and ventricular pacing leads during RF ablation close to the atrial lead even when the pacemaker was set to pace asynchronously. We also explain the mechanism behind this unusual pacemaker response.

## Introduction

Catheter ablation in patients with cardiovascular implantable electronic devices (CIEDs) is commonplace in contemporary practice. Because CIEDs are susceptible to electromagnetic interference (EMI) during catheter ablation and the effects of EMI on CIEDs can be variable and unpredictable, an awareness of the spectrum of responses of CIEDs to EMI, including uncommon ones, is key in being able to plan for a safe ablation procedure. In this report, we describe a case where a dual-chamber pacemaker set to pace asynchronously failed to deliver atrial and ventricular pacing pulses to the myocardium during radiofrequency (RF) ablation near the tip of the atrial lead, resulting in the failure of the pacemaker to capture both atrial and ventricular myocardium.

## Case presentation

**Figure 1:**
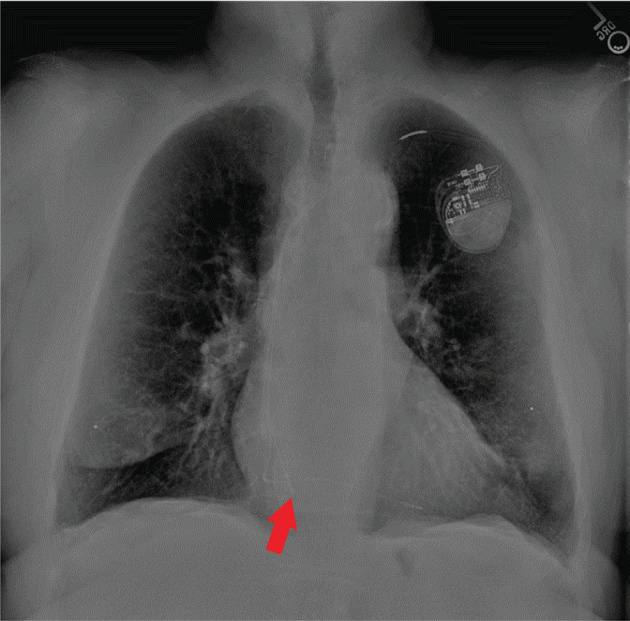
Chest radiograph showing a dual-chamber pacemaker, with the tip of a chronically dislodged atrial lead (red arrow) on the floor of the right atrium.

A 73-year-old man underwent cavotricuspid isthmus (CTI) ablation for symptomatic typical atrial flutter. He also had complete heart block and a dual-chamber pacemaker (pulse generator Ingenio model K174, right atrial [RA] lead Dextrus model 4136, and right ventricular [RV] lead Dextrus model 4137; Boston Scientific, Marlborough, MA, USA) that was implanted 9 years prior to the ablation procedure. The RA lead had been chronically dislodged, with its tip located on the floor of the RA **(Figure 1)**. A review of his medical records indicated that it had become dislodged within 3 months of implantation, but it had maintained excellent sensing and pacing characteristics through the years and no intervention was performed to reposition it. A recent pacemaker interrogation while he was in sinus rhythm and using a bipolar sensing and pacing configuration showed a P-wave amplitude of 2.2 mV, an atrial pacing threshold of 0.9 V @ 0.4 ms, an atrial pacing impedance of 525 Ω, and a low frequency of atrial pacing (<5%).

**Figure 2:**
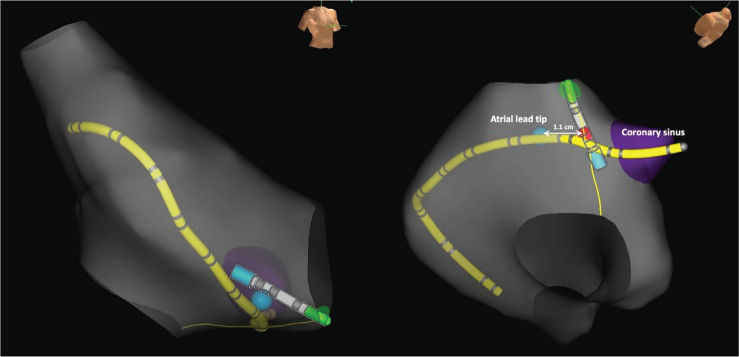
Right anterior oblique and left anterior oblique caudal views of the right atrium, together with a 20-pole catheter and an ablation catheter. The tip of the atrial lead was tagged using a blue dot. A line of ablation was performed medially (yellow line), at least 1.1 cm away from the atrial lead tip.

**Figure 3:**
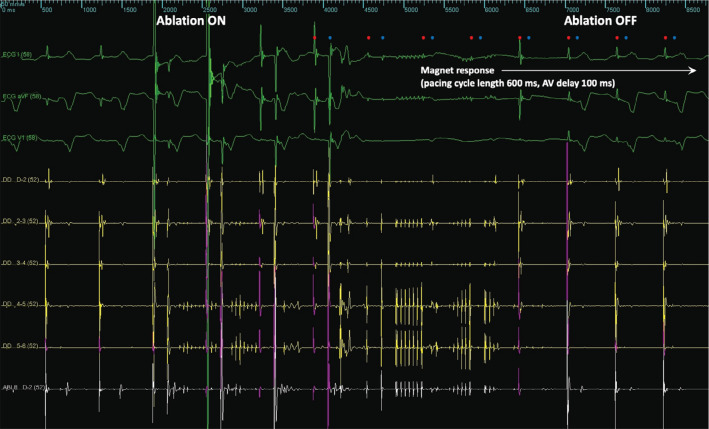
Loss of atrial and ventricular capture during radiofrequency ablation. A magnet was placed over the pacemaker to make it pace asynchronously during ablation. A magnet response can be seen from the shortening of the pacing cycle length from 667 ms (rate of 90 pulses/min) on the left side of the tracing to 600 ms (rate of 100 pulses/min) on the right and shortening of the atrioventricular delay from 180 ms on the left to 100 ms on the right. The red and blue dots represent the timing (or anticipated timing) of atrial pacing and ventricular pacing, respectively. The second pair of red and blue dots coincides with artifacts that may have been generated by sense channel input blanking at the time pacing pulses were being issued by the pulse generator and/or shunting of the current from the pacing pulses to the pacemaker case, rather than actual delivery of pacing current into the leads and the myocardium. *Abbreviation:* AV, atrioventricular.

On the day of ablation, the patient was in sinus rhythm. Fluoroscopic imaging indicated that the RA lead tip was in the mid-portion of the CTI. A 20-pole catheter was looped inside the RA and its tip was positioned inside the coronary sinus. The location of the RA lead tip was tagged in the 3-dimensional electroanatomic mapping system (EnSite Precision™; Abbott, Chicago, IL, USA), and a decision was made to create a medial CTI ablation line at least 1.1 cm medial to the RA lead tip **(Figure 2)**. A non-irrigated 8-mm-tip RF ablation catheter was used, with a power output of 70 W, a temperature limit of 60°C, and a duration of 30 s for every lesion. The ground pad was placed at the lower back. With the pacemaker pacing the atrium and ventricle sequentially in DDD mode, the first application of RF energy on the tricuspid annulus resulted in ventricular asystole. Ablation was stopped immediately, and the paced QRS complexes returned. The initial thought was that EMI from ablation caused inhibition of pacing, so the pacemaker was set to pace asynchronously, eg, by programming it to DOO or Electrocautery Protection Mode or by placing a magnet over it. Despite this, ventricular asystole kept occurring at the start of every ablation. A closer inspection of the tracings made it apparent that the pacemaker actually never ceased to issue pacing stimuli during ablation. During the period of ventricular asystole, one could march out atrial and ventricular pacing stimuli, as anticipated on the basis of the programmed lower rate limit and atrioventricular delay or the expected magnet response, all the way until the paced QRS complexes returned. During this same period, artifacts that looked like pacing artifacts sometimes appeared at the anticipated timing of atrial and ventricular pacing. Therefore, it was myocardial capture in both chambers, rather than the creation of pacing pulses by the pacemaker, that ceased with ablation and resumed with discontinuation of ablation **(Figure 3)**. A quadripolar catheter was then advanced into the RV and used for pacing throughout the rest of the ablation.

We proceeded with ablation using the same ablation catheter and the same ablation settings. The ablation catheter was maneuvered carefully to make sure it did not come into contact with the RA lead during creation of any of the lesions. After every lesion that was placed 1.1–1.2 cm away from the RA lead tip, the RA lead parameters were checked using the programmer, and significant changes were observed. The P-wave amplitude dropped to as low as 0.5 mV and the atrial pacing threshold increased to as high as 2.8 V @ 0.4 ms. These improved within 1 min of completing the lesion. The atrial pacing impedance was also noted to sometimes drop transiently to <200 Ω, normalizing within 1 min of completing the lesion. Because the patient had good sinus node function and required minimal atrial pacing and because atrial sensing remained intact, a decision was made to complete the medial CTI ablation line despite the changes observed in the RA lead parameters during ablation. Bidirectional block across the CTI was achieved and persisted through a 30-min waiting period. At the end of the procedure, the RA lead parameters were as follows: P-wave amplitude, 1.1 mV; atrial pacing threshold, 2.0 V @ 0.4 ms; and atrial pacing impedance, 500–600 Ω. The RA pacing output and sensitivity were adjusted to ensure adequate safety margins. The RV lead did not exhibit any significant changes in parameters before and after ablation. Two months later, the RA lead parameters were as follows: P-wave amplitude, 1.3 mV; atrial pacing threshold, 2.2 V @ 0.4 ms; and atrial pacing impedance, 530 Ω.

## Discussion

RF ablation generates an alternating current in the frequency range of 500–1,000 kHz and can be a source of EMI for CIEDs.^1,2^ In many cases, this type of EMI results in oversensing by the CIED, which in turn can lead to a variety of responses.

These include pacing inhibition, rapid tracking in dual-chamber systems, spurious tachyarrhythmia detection, mode switch to an asynchronous pacing mode (noise reversion mode) or a non-tracking mode (eg, DDI), extension or resetting of the refractory period resulting in functional undersensing and asynchronous pacing, and inappropriate therapy (anti-tachycardia pacing or shock). In pacemakers where the rate response to a minute-ventilation sensor is on, EMI from RF ablation can activate the sensor and result in pacing at the upper sensor rate.^3^ Rarely, an electrical reset occurs whereby operating parameters are re-programmed, eg, to recommended replacement time or elective replacement interval parameters or to power-on reset parameters. In addition to eliciting these behaviors from CIEDs, ablation close to the tip of a pacing lead can cause alterations in the lead–tissue interface, which can impact the pacing and sensing characteristics of the lead. In extreme cases, scar in the lead–tissue interface can result in exit block with pacing.

The unusual pacemaker behavior elicited by RF ablation in this case was the failure of pacing stimuli to capture both atrial and ventricular myocardium, which we believe was due to the operation of a high-voltage protection circuit in the pacemaker and shunting of current from the pacemaker away from the leads. On the recording system, this gave the appearance of ventricular asystole in this patient with complete heart block despite setting the pacemaker to pace asynchronously (eg, by programming the device in DOO or Electrocautery Protection mode or applying a magnet to activate the Hall-effect sensor). Rare descriptions of this behavior during catheter ablation have been reported with older generations of pacemakers from Intermedics (Freeport, TX, USA), Medtronic (Minneapolis, MN, USA), Vitatron (Kyle, TX, USA), and Telectronics (Englewood, CO, USA).^4–7^

Before a signal from a lead enters the sense amplifier in the internal circuitry of contemporary pacemakers, it typically goes through feedthrough filter capacitors that filter out high-frequency EMI in the megahertz range, such as signals from mobile phones. It also goes through a proprietary high-voltage protection circuit or a surge-suppressor that contains elements such as Zener diodes or thyristors that help protect the internal circuitry of the pacemaker from permanent damage in the presence of strong electromagnetic fields. When the high-voltage protection circuit detects excessive voltage (eg, >10 V) at the lead tip, it acts as a short circuit and shunts the current away from the internal circuitry, eg, toward the pacemaker case. As a result, the sense amplifier will not see the signal at the lead tip. At that same instant, any pacing stimulus issued by the output pulse generator of the internal circuitry will likely be shunted by the high-voltage protection circuit toward the case as well and will not be delivered to the pacing electrode. If telemetered signals in a programmer were to be monitored during this time, annotations for atrial and/or ventricular pacing may be seen, but none of the pacing stimuli will be delivered to the lead or myocardium. This phenomenon is not the same as pacing inhibition, where a pacing pulse is not even issued by the output pulse generator. In this case, signals that looked like pacing stimuli sometimes appeared, with varying amplitudes, in the recording system even though the pacing current was being shunted away from the leads (eg, the second pair of red and blue dots in **Figure 3**). We believe that this artifact was generated by sense channel input blanking (ie, transient disconnection and reconnection of a sense channel input to the lead) at the time a pacing pulse was being issued by the output pulse generator and/or shunting of current from the pacing pulse to the pacemaker case, which was in close proximity to the electrocardiogram electrodes.

In pacemakers where multiple electrodes are present, depending on the manufacturer, the effect of excessive voltage near one electrode may or may not affect what happens to the other electrodes. In this case, activation of the high-voltage protection circuit affected both the atrial and ventricular channels, even though ablation was confined to the atrium and occurring far from the ventricular lead tip **(Figure 3)**. This argues against the exit block from the atrial lead tip as the sole or main mechanism behind the failure to capture atrial myocardium during ablation.

Another type of high-voltage protection circuit, reported in an old pacemaker model (Telectronics model 1254), relies on the opening of the pacemaker’s output switches when a high voltage is detected.^5,6^ This prevented retrograde currents that were flowing up the lead from entering the pacemaker. Because this effectively disconnected all electrodes and the generator case from the output pulse generator, any pacing stimulus at this instant would be unable to conduct down the pacing leads to capture the myocardium, even in the DOO or VOO mode. When the high voltage was no longer detected, the switches would close and current could once again leave the generator. Some manufacturers of contemporary implantable cardioverter-defibrillators employ this mechanism of switches for high-voltage protection.

During RF ablation, the magnitude of current induced at the tip of a pacing lead has been shown to be inversely related to the distance of the ablation site from the tip, explaining the increased risk of EMI when ablation is performed within 1–2 cm of the lead tip.^4,8,9^ Accordingly, ablation close to a lead tip is more likely to trigger the high-voltage protection circuit of a pacemaker and may not cause “appropriate” pacing inhibition or switching to a noise-reversion mode for a pacemaker originally set to the DDD or VVI mode in a way that oversensed signals typically do.

Finally, the changes observed in the RA lead parameters during ablation in this case—specifically, the decrease in P-wave amplitude, the increase in pacing threshold, and the transient drop in pacing impedance—were reflective of alterations in the lead–tissue interface as thermal myocardial lesions were created near the lead tip, rather than direct damage to the lead or its insulation, as the ablation catheter tip never came in contact with the lead at any point during ablation. In cases where movement of the ablation catheter can result in intermittent contact with the lead, the use of a long sheath can be considered to stabilize the catheter and keep it consistently away from the lead. Alternatively, a snare can be used to pull the lead away from the ablation catheter.^10^

## Conclusion

The response of a pacemaker to RF ablation can be variable and unpredictable. One uncommon response is the failure of the pacemaker to deliver its pulses to the lead and the myocardium when EMI activates a high-voltage protection circuit and the current from the output pulse generator is shunted away from the lead. This can happen even when the pacemaker is set to pace asynchronously. When this occurs in a pacemaker-dependent patient, an external pacing catheter will be required to complete the ablation safely.
